# Knowledge of Human Papillomavirus (HPV), Attitudes and Practices Towards Anti-HPV Vaccination Among Israeli Pediatricians, Gynecologists, and Internal Medicine Doctors: Development and Validation of an Ad Hoc Questionnaire

**DOI:** 10.3390/vaccines7040157

**Published:** 2019-10-21

**Authors:** Rola Khamisy-Farah, Mohammad Adawi, Haneen Jeries-Ghantous, Jacob Bornstein, Raymond Farah, Nicola Luigi Bragazzi, Marwan Odeh

**Affiliations:** 1 Clalit Health Service, Akko, Azrieli Faculty of Medicine, Bar-Ilan University, Safed 13100, Israel; rkhamisy@yahoo.com; 2 Department of Medicine, Baruch Padeh Medical Center, Azrieli Faculty of Medicine, Bar Ilan University, Ramat Gan 5290002, Israel; adawimo1802@gmail.com; 3 Department of Obstetrics and Gynecology, Galilee Medical Center, Nahariya 22100, Galilee, Israel; haneenj90@gmail.com (H.J.-G.); marwan20@bezeqint.net (M.O.); 4 Faculty of Medicine, Bar Ilan University, Safed 13100, Israel; 5 Department of Internal Medicine B, Ziv Medical Center, Safed-Israel, Azrieli Faculty of Medicine, Bar-Ilan University, Safed 13100, Israel; Jacobb@gmc.gov.il (J.B.); raymond.f@ziv.health.gov.il (R.F.); 6 Department of Mathematics and Statistics, Laboratory for Industrial and Applied Mathematics (LIAM), York University, Toronto, ON M3J 1P3, Canada

**Keywords:** human papillomavirus (HPV), knowledge, attitudes, and practices (KAP) questionnaire, development and validation of questionnaire, psychometric properties, vaccine hesitancy, pediatricians, gynecologists, and internal medicine doctors, Israel

## Abstract

Human papillomavirus (HPV) is a highly widespread virus which is responsible for one of the most common sexually transmitted infections. Two main preventative strategies exist: anti-HPV vaccination and cervical screening. Health-care workers play a key role in promoting public health campaigns; however, vaccine hesitancy is an often under-recognized challenge. To investigate the overall knowledge of HPV and HPV-related issues, as well as the attitudes and practices of health professionals towards recommending the anti-HPV vaccine, an ad hoc knowledge, attitudes, and practices (KAP) questionnaire was developed and validated in a sample of 139 Israeli pediatricians, gynecologists, and internal medicine doctors. The KAP questionnaire was found to be psychometrically valid and sound (with an r_KR-20_ coefficient of 0.74 for the second part and a Cronbach’s alpha of 0.85 for the third part). Furthermore, the present study confirmed the importance of health-care providers in recommending the immunization practice. Parents that had been strongly advised by health-care providers to vaccinate their children accepted immunization for their girls (odds ratio (OR) 1.09 (95% CI 1.04–1.14)) and boys (OR 1.06 (95% CI 1.02–1.10)), had a lower probability of deciding to postpone the immunization appointment (OR 0.81 (95% CI 0.66–0.98)), had fewer doubts and concerns about the vaccine (OR 0.69 (95% CI 0.54–0.89)), and had a lower probability of refusing the vaccination (OR 0.93 (95% CI 0.86–0.99)). Interestingly, the use of new, emerging tools such as ad hoc websites, applications, and other interactive devices reduced vaccine hesitancy (OR 0.90 (95% CI 0.82–0.99)) and concerns about the side-effects of the vaccine (OR 0.92 (95% CI 0.86–0.99)). However, among Israeli health-care workers, knowledge was generally moderate, with updated information lacking in about 30% of surveyed health-care providers and approximately 20% of them not recommending the anti-HPV vaccine among boys. This study has practical implications for policy- and decision-makers in that they should be aware of the overall level of knowledge among health-care workers and should implement ad hoc educational interventions to address gaps in knowledge and help medical providers routinely recommend the anti-HPV vaccine both to male and female children and adolescents.

## 1. Introduction

Human papillomavirus (HPV), a nonenveloped DNA virus, is a highly widespread and common pathogen, which is responsible for one of the most common sexually transmitted infections [1]. It generally causes benign lesions (including warts and papillomas), but if not spontaneously cleared or properly managed and treated, it can lead to malignant ones, such as intraepithelial lesions and neoplasia [2,3,4,5]. Indeed, there exist many strains of HPV—over 200—with some types conferring a high risk of developing cancers (the so-called oncogenic strains) [2]. 

Globally, approximately 4.5% of all malignancies can be attributed to HPV (8.6% among women and 0.8% among men), with HPV being the main etiopathogenetic factor of up to 83% of all cervical cancers [6]. Other HPV-attributable cancers include vaginal, vulvar, anal, penile, and head-and-neck malignancies [7,8]. 

Specifically in Israel, HPV generates a dramatic burden, even though this is partially mitigated by cultural and religious factors (including male circumcision and religious creeds, such as the Jewish or the Islamic faiths, which strongly regulate sexual behavior) [9]. In this country, approximately 3 million women aged 15 years and older are at risk of being infected by HPV and, consequently, developing cervical cancer. According to a survey based on the computerized system of the second largest Israeli health maintenance organization, the “Maccabi Healthcare Services”, 737 and 3459 cases of cervical cancer and cervical intraepithelial neoplasia grade III (CIN3) were reported, respectively, in the period 1986–2010, with an annual age-adjusted incidence rate of cervical cancer of 3.7 per 100,000 in 2010, which is a significant increase with respect to 1986 (1.6 per 100,000, *p*-value for trend = 0.0001). Similarly, the annual age-adjusted incidence rate of CIN3 ranged from 3.9 per 100,000 in 1986 to 40.4 per 100,000 in 2010 (*p*-value for trend = 0.0001) [10]. 

Anti-HPV vaccination and gynecological screening (the Papanicolaou smear or Pap test) represent two major preventative strategies to counteract or, at least, mitigate such a burden, the former being an approach of primary prevention, and the latter being a strategy of secondary prevention [11,12]. 

Currently, three types of anti-HPV vaccine exist: a bivalent preparation (traded as Cervarix^©^, against HPV types 16 and 18), a quadrivalent one (marketed as Gardasil^©^, targeting HPV types 16, 18, 31, and 33), and finally, a nonavalent one (traded as Gardasil-9^©^, against HPV types 6, 11, 16, 18, 31, 33, 42, 52, and 58) [11,12,13]. 

While being generally administered among females, recently, the American Academy of Pediatrics (AAP) has recommended the use of anti-HPV vaccines also among males [14]. Various high-quality randomized controlled trials (RCTs), such as the Patricia, Future I, and Future II studies, have shown the effectiveness of anti-HPV vaccination [15,16], and several investigations and RCTs, including the Artistic, Swedescreen, Pobascam, and NTCC studies, have demonstrated the clinical usefulness of cervical screening [17,18,19,20,21,22,23]. 

In 2013, in Israel, a universal program for HPV vaccination was introduced for eighth-grade girls at middle schools and for ninth-grade girls at health bureaus [24]. In greater detail, the Cervarix^©^ vaccine was added to the health basket, that is, the basket of drugs and medical treatments that are subsidized by the government. This enabled achieving an overall coverage rate of approximately 60%, being higher among secular Jews and Arabs and lower among Orthodox Jews. In 2015, the immunization program was further expanded, introducing the anti-HPV vaccination (Gardasil^©^) also for men and boys aged between 9 and 26 years. For the year 2019–2020, the Gardasil-9^©^ vaccine is being provided both to eighth-grade girls and boys at schools. According to the Israeli Ministry of Health’s guidelines [24], cervical screenings are recommended every three years and, currently, the coverage rate is rather low, being approximately 32% (that is to say, one out of three women aged 21–59 years is compliant with the recommendations). 

Health-care providers are essential in disseminating high-quality and scientific-evidence-based information on HPV and the HPV-related burden. A recent state-wide survey carried out in Minnesota, United States, found that anti-HPV vaccine hesitancy among health-care professionals may discourage vaccine uptake among children and adolescents [25]. However, there is a dearth of information concerning the knowledge of HPV, as well as the attitudes and practices towards anti-HPV vaccination among Israeli pediatricians, gynecologists and internal medicine physicians, who are supposed to recommend the immunization practices to boys and girls. 

Therefore, the present cross-sectional survey-based study was designed to address this gap in knowledge.

## 2. Materials and Methods 

### 2.1. Ethical Clearance

The study protocol of the present investigation was reviewed in depth and, being questionnaire based, received an exemption from the Helsinki Commission of the Ziv Medical Center, Safed, Israel. 

### 2.2. Development and Validation of the Ad Hoc Knowledge, Attitudes, and Practices (KAP) Questionnaire

A KAP questionnaire concerning knowledge of HPV, as well as attitudes and practices towards anti-HPV vaccination, was developed based on (i) an extensive literature search; (ii) a focus group with a selected number of Israeli pediatricians, gynecologists, and internal medicine doctors; (iii) a revision from a panel of experts and scholars; and, finally, (iv) a pilot test in a sample of 20–30 subjects. 

The final form of the questionnaire was administered to pediatricians, gynecologists, and internal medicine doctors of the Galilee Medical Center in Nahariya and of the Ziv Hospital in Safed, Israel. 

The questionnaire consisted of three parts: a sociodemographic part with questions concerning age, gender, marital status, religion, profession, clinical ward attended, and years of experience; a part regarding knowledge of HPV and HPV-related burden; and a part related to attitudes and practices towards anti-HPV vaccination. More in detail, the third part of the questionnaire was theoretically built to consist of two subsets of items: the first subset concerned the awareness of the health-care provider about the importance, safety, and effectiveness of the anti-HPV vaccination and his/her attitude towards strongly recommending it by establishing a relationship and alliance with the child and his/her family. The second subset of items regarded vaccine counseling and the use of new, emerging tools, such as ad hoc vignettes, cartoons, websites, applications, and other interactive devices.

### 2.3. Statistical Analysis

Before proceeding with data handling and statistical processing, figures were visually inspected to capture potential outliers. Continuous variables were expressed as mean ± standard deviation, whereas categorical parameters were computed as percentages, where appropriate. 

The validity of the second part was computed by calculating the Kuder–Richardson Formula 20 (KR-20) coefficient (r_KR-20_) [25,26,27,28], utilizing the following formula:

(1) $$r_{KR-20}=\frac{K}{K-1}\cdot \left(1-\frac{\sum_{i=1}^{K}p_{i}\cdot q_{i}}{\sigma_{X}^{2}}\right)$$

where *K* is the number of items, $p_{i}$ is the percentage of right replies to item *i*, $q_{i}$ is the percentage of wrong replies to item *i*, and $\sigma_{X}^{2}$ is the variance of the total observed test scores, computed as:

(2) $$\sigma_{X}^{2}=\frac{\sum_{i=1}^{n}{\left(X_{i}-\bar{X}\right)}^{2}}{n}$$

where *n* is the sample size. The KR-20 represents a particular case of the Cronbach’s alpha for items that imply a dichotomous reply (yes versus no, right versus wrong). Unlike the Kuder–Richardson Formula 21 (KR-21), the KR-20 makes the more realistic assumption that the test items differ in terms of difficulty (with some questions being easier, some being more difficult). Therefore, different variables, such as the spread among the scores, the presence of some particularly challenging questions, and the length of the questionnaire, could impact on the r_KR-20_.

The validity of the third part was computed calculating the Cronbach’s alpha coefficient, using the following formula [29]:

(3) $$\alpha =\frac{K}{K-1}\cdot \left(1-\frac{\sum_{i=1}^{K}\sigma_{Y_{i}}^{2}}{\sigma_{X}^{2}}\right)$$

where $\sigma_{Y_{i}}^{2}$ is the variance of the component *i* for the current sample of subjects.

The magnitude of the Cronbach’s alpha coefficient was interpreted based on the following rule of thumb: unacceptable if less than 0.5, poor in the range of 0.5–0.6, questionable in the range of 0.6–0.7, acceptable in the range of 0.7–0.8, good in the range of 0.8–0.9, and excellent if equal to or greater than 0.9.

Both the r_KR-20_ and the Cronbach’s alpha coefficient can be standardized as follows:

(4) $$r_{KR-20}/\alpha_{standardized}=\frac{K\cdot \bar{r}}{1+\left(K-1\right)\cdot \bar{r}}$$

where $\bar{r}$ is the average of the nonredundant correlation coefficients (in other words, the upper or lower triangle of the correlation matrix).

A logistic regression model was conducted to shed light on the determinants of overall knowledge of HPV and HPV-related issues, the attitudes and practices towards recommending the anti-HPV vaccination, as well as their impact on parents’ intention to vaccinate their children. According to the Hosmer–Lemeshow procedure, only covariates with a *p*-value less than 0.25 at the univariate analysis were entered in the models. Gender and age, being potential confounders, were included into the regression model. The goodness of fit of the model was verified performing the Hosmer–Lemeshow test. 

Finally, the impact on parents’ intention to vaccinate or not their children was assessed by means of odds ratios (ORs) computed together with their 95% confidence interval (CI).

All statistical analyses were performed by means of the commercial “Statistical Package for Social Sciences” (SPSS) version 24.0 for Windows (IBM, Armonk, NY, USA). For all statistical analyses, figures with *p*-values less than 0.05 were considered statistically significant. 

## 3. Results

### 3.1. Recruited Sample

A sample of 139 health-care workers took part in the present study (average age 31 years (95% inter-quartile range (IQR) 29–47), 61.2% females and 38.8% males, 53.2% married, 68.3% specialists/doctors and 31.7% residents, 64.0% with less than 5 years of experience, and 37.4% of Jewish creed). Further characteristics of the sample are shown in Table 1.

### 3.2. Development and Validation

The items of the questionnaire are presented in Supplementary Material 1. The validity of the second part was satisfactory (r_KR-20_ coefficient of 0.74, standardized coefficient adjusted for the number of items of 0.75). Dropping one item per time generally resulted in a decreased internal consistency coefficient (except for item one), confirming the validity of the questionnaire. The third part displayed an overall good internal consistency (Cronbach’s alpha coefficient 0.85). For further information, the reader is referred to Table 2 and Table 3.

Specifically concerning the third part of the questionnaire, the first subset of items had a satisfactory internal consistency of 0.776, whereas the second subset had a validity of 0.841 (Table 3).

### 3.3. Level of HPV-Related Knowledge

Overall, HPV-related knowledge resulted in a mean score of 7 (95% IQR 5–8), with the percentage of right replies ranging from 35.3% (for item 1, “frequency of gynecological examinations”) to 87.1% (for item 3, “preventative strategies for cervical cancer”), as reported in Table 4. Percentages of right and wrong replies were not statistically significant for test items 4 (“Israeli Ministry of Health’s guidelines for anti-HPV vaccination”), 6 (“number of HPV strains related to cervical cancer and other malignancies”), and 7 (“body regions that can be infected by HPV”). Except for item 1, for the remaining items, percentages of right replies were significantly higher than percentages of wrong replies. In terms of determinants, no differences could be found in terms of age, gender, marital status, profession, years of experience, and religious creed. 

### 3.4. Attitudes Towards Recommending Anti-HPV Vaccination and Their Determinants

The mean score of the third part of the questionnaire was 3.34 ± 0.41. Average scores for the first and second subsets were 3.32 ± 0.37 and 3.39 ± 0.75, respectively. Correlation between the first and the second subsets was 0.47 (95% CI 0.32–0.59; *p* < 0.0001). 

The score of the first subset was found to be higher in physicians operating in the pediatric ward (OR 6.40 (95% CI 1.26–32.48)), whereas no significant predictor was associated with the score of the second subset (Table 5).

Most health-care workers recommended anti-HPV vaccination among girls (98.6%), whereas only 79.9% of them recommended the immunization practice among boys. 

### 3.5. Impact on Parents’ Intention to Vaccinate Their Children

Parents that had been strongly advised by health-care providers to vaccinate their children accepted immunization for their girls (OR, 1.09 (95% CI 1.04–1.14)) and boys (OR 1.06 (95% CI 1.02–1.10)), had a lower probability of deciding to postpone the immunization appointment (OR 0.81 (95% CI 0.66–0.98)), had fewer doubts and concerns about the vaccine (OR 0.69 (95% CI 0.54–0.89)), and had a lower probability of refusing the vaccination (OR 0.93 (95% CI 0.86–0.99)). Interestingly, the use of new, emerging tools such as ad hoc websites, applications, and other interactive devices reduced vaccine hesitancy (OR 0.90 (95% CI 0.82–0.99)) and concerns about side-effects of the vaccine (OR 0.92 (95% CI 0.86–0.99)). 

## 4. Discussion

Anti-HPV vaccination and HPV testing, such as the Pap test, represent, respectively, a safe, effective immunization practice and a major secondary preventative strategy that can reduce HPV-related burden. Health-care providers play a key role in advising parents to vaccinate their children in terms of trust and being perceived of as authoritative, highly reliable sources of information. Indeed, some studies have shown that parents who had been very strongly recommended to have their son immunized had higher odds of vaccinating their children compared with parents who were not very strongly recommended [30]. It is therefore of crucial importance to investigate the levels of HPV-related knowledge among health-care workers (such as pediatricians, gynecologists, and internal medicine doctors) and the determinants and predictors of attitudes towards anti-HPV vaccination. 

Our findings indicate that knowledge of HPV and HPV-generated clinical burden is moderate among health-care workers in Israel. These results are in line with the existing scholarly literature. In Greece, several gaps in knowledge among female health-care providers could be detected, especially in terms of updated information on cervical cancer prevention through HPV testing and vaccination, with only 80% of them being aware of the existence of cervical screenings [31]. In Spain, a sample of pediatricians and pediatric nurses was surveyed and both general and specific vaccine knowledge was tested. The authors found a lack of adequate knowledge in up to 40% of the health-care professionals surveyed, with responses concerning anti-HPV vaccination being significantly associated with the highest degree of doubts [32].

However, in our study, we could not find differences in terms of level of HPV-related knowledge between residents and specialists/doctors as well as among gynecologists, pediatricians, and internal medicine doctors (except for the first subset of items of the third part of the questionnaire), whereas some scholars have reported differences, with the highest level of knowledge of and belief in the anti-HPV vaccine, its effectiveness, and its safety being observed among pediatricians and school medicine specialists [33]. On the other hand, a study conducted in Louisiana, United States failed to find a significant association between knowledge strength and levels and years in practice or practice type [34]. 

Furthermore, in Israel, the decision to also include boys in the immunization program ignited a rather intense debate, given the relationship between the vaccine and sexual behaviors [35,36]. Concerning the attitude to recommend anti-HPV vaccine among boys, in our study, only 20% of health-care providers did not recommend the immunization practice, a percentage which is much lower than other percentages found by scholars in other countries. In Italy, for instance, in a national online cross-sectional survey carried out among primary care physicians, approximately 82% of them would not recommend parents to vaccinate their male children [37]. However, our finding is in line with the results obtained by other scholars in Canada, where 79.0% and 75.8% of male physicians recommended the immunization against HPV to male patients and male partners of female patients, respectively [38]. 

In Israel, several parents are hesitant to vaccinate their sons [39,40] due to cultural and religious beliefs. In our study, we found that parents that had been strongly advised by medical providers to vaccinate their children were less reluctant to postpone or to refuse to immunize their sons, being more convinced of the safety and effectiveness of the vaccine. Given the important role played by health-care providers in promoting the anti-HPV vaccination campaign [41,42], it is crucial to have constantly educated and updated health professionals, who can have a positive impact on parents’ intention to vaccinate their children, as well as to have a psychometrically sound instrument that can quantitatively assess knowledge, beliefs, attitudes, and practices among health-care workers.

Vaccine hesitancy among health-care professionals is an under-recognized public health concern that may jeopardize the success of immunization programs and needs, as such, to be properly addressed [43]. Health policy- and decision-makers should be aware of this phenomenon and should implement a series of educational interventions, such as continuing medical education, aimed at helping medical providers to be constantly updated and routinely recommending the anti-HPV vaccine both to female and male children and adolescents [44]. 

However, the present investigation is not without limitations, including the small sample size employed and its cross-sectional study design. Longitudinal, prospective studies verifying, for instance, the effect of programs of continuing education and utilizing random stratified sampling techniques, in order to ensure the generalizability of the findings, are warranted. 

## 5. Conclusions

The KAP questionnaire was found to be psychometrically valid and reliable and can be used, as such, by other scholars to investigate knowledge of HPV, as well as attitudes and practices towards anti-HPV vaccination. Among Israeli pediatricians, gynecologists, and internal medicine doctors, knowledge was generally moderate, with updated information lacking in about 30% of surveyed health-care providers and approximately 20% of them not recommending the anti-HPV vaccine among boys. On the other hand, a good level of knowledge and positive attitudes towards recommending the anti-HPV vaccine significantly impacted on parents’ intention to vaccinate their children. This study has practical implications for policy- and decision-makers in that they should be aware of the overall level of knowledge among health-care workers and their attitudes towards recommending the anti-HPV vaccine. Continuing medical education and other educational interventions could be an effective strategy to keep health professionals constantly updated and to potentially counteract vaccine hesitancy among health-care workers. 

## Figures and Tables

**Table 1:** Major characteristics of the sample recruited and included in the present study.

Variable	Value
Age (mean ± standard deviation; median)	37.66 ± 12.41; 31
Gender (number; percentage)	
Female	85 (61.2%)
Male	54 (38.8%)
Marital status (number; percentage)	
Not married	65 (46.8%)
Married	74 (53.2%)
Profession (number; percentage)	
Resident	44 (31.7%)
Specialist/doctor	95 (68.3%)
Years of experience (number; percentage)	
Less than 5 years	89 (64.0%)
More than 5 years	50 (36.0%)
Clinical ward (number; percentage)	
Pediatrics	15 (10.8%)
Gynecology	37 (26.6%)
Internal medicine	87 (62.6%)
Religious creed (number; percentage)	
Jewish	52 (37.4%)
Christian	32 (23.0%)
Muslim	38 (27.3%)
Druze	12 (8.6%)
Other	5 (3.6%)

**Table 2:** Reliability statistics of the second part of the questionnaire according to the Kuder–Richardson Formula 20 (KR-20).

Variable Dropped	r_KR-20_	Change	r_KR-20_ (Standardized and Adjusted)	Change
Item 1	0.7502	0.008873	0.7538	0.007909
Item 2	0.7162	−0.02517	0.7217	−0.02423
Item 3	0.7189	−0.02245	0.7199	−0.02599
Item 4	0.7110	−0.03032	0.7194	−0.02647
Item 5	0.7133	−0.02803	0.7189	−0.02702
Item 6	0.7238	−0.01754	0.7293	−0.01657
Item 7	0.7022	−0.03918	0.7113	−0.03461
Item 8	0.7055	−0.03583	0.7119	−0.03405
Item 9	0.7325	−0.008852	0.7344	−0.01155
Item 10	0.7277	–0.01367	0.7300	–0.01592

**Table 3:** Reliability statistics of the third part of the questionnaire.

Item	Mean	Standard Deviation	Cronbach’s Alpha if Item Dropped
Item 1	4.36	0.96	0.848
Item 2	3.85	1.29	0.851
Item 3	1.71	0.96	0.862
Item 4	2.10	1.24	0.865
Item 5	2.91	1.42	0.853
Item 6	3.27	1.40	0.855
Item 7	3.51	1.48	0.853
Item 8	3.40	1.13	0.855
Item 9	4.00	0.92	0.849
Item 10	4.38	0.91	0.849
Item 11	4.30	0.96	0.849
Item 12	4.43	0.81	0.848
Item 13	2.92	1.05	0.849
Item 14	3.62	1.00	0.852
Item 15	2.89	1.25	0.856
Item 16	3.53	1.00	0.844
Item 17	3.35	1.07	0.844
Item 18	3.60	0.95	0.845
Item 19	3.47	1.02	0.844
Item 20	3.65	0.89	0.843
Item 21	3.55	0.93	0.843
Item 22	2.72	0.98	0.845
Item 23	2.71	0.97	0.844
Item 24	3.01	0.97	0.846
Item 25	3.02	0.96	0.846
Item 26	3.16	0.86	0.846
Item 27	3.12	0.90	0.845
Item 28	3.21	1.01	0.851
Item 29	3.24	1.02	0.852
Item 30	2.54	1.01	0.851
Item 31	2.57	0.99	0.850
Item 32	4.03	0.95	0.844
Item 33	3.46	1.19	0.841
Item 34	4.08	1.00	0.844
Item 35	3.61	1.34	0.842
Item 36	2.93	1.14	0.847
Item 37	3.54	1.07	0.859
Item 38	3.33	1.16	0.842
Item 39	3.64	1.20	0.842
Item 40	3.36	1.25	0.844
Item 41	2.82	1.35	0.848
Item 42	3.40	1.24	0.844
Item 43	3.29	1.23	0.844

**Table 4:** Frequency of wrong and right replies for the second part (“level of HPV-related knowledge”) of the questionnaire.

Item	Number (Percentage)	*p*-Value
Item 1 (frequency of gynecological examinations)		0.015
Wrong reply	90 (64.7%)	
Right reply	49 (35.3%)	
Item 2 (to whom the Pap test is recommended)		0.022
Wrong reply	50 (36.0%)	
Right reply	89 (64.0%)	
Item 3 (preventative strategies for cervical cancer)		<0.001
Wrong reply	18 (12.9%)	
Right reply	121 (87.1%)	
Item 4 (Israeli Ministry of Health’s guidelines for anti-HPV vaccination)		0.905
Wrong reply	71 (51.1%)	
Right reply	68 (48.9%)	
Item 5 (number of HPV strains)		<0.001
Wrong reply	28 (20.1%)	
Right reply	111 (79.9%)	
Item 6 (number of HPV strains related to cervical cancer and other malignancies)		0.338
Wrong reply	78 (56.1%)	
Right reply	61 (43.9%)	
Item 7 (body regions that can be infected by HPV)		0.187
Wrong reply	58 (41.7%)	
Right reply	81 (58.3%)	
Item 8 (diseases against which the anti-HPV vaccination offers protection)		0.000
Wrong reply	39 (28.1%)	
Right reply	100 (71.9%)	
Item 9 (percentage of girls aged 15–18 years being sexually active)		0.000
Wrong reply	31 (22.3%)	
Right reply	108 (77.7%)	
Item 10 (percentage of boys aged 15–18 years being sexually active)		<0.001
Wrong reply	19 (13.7%)	
Right reply	120 (86.3%)	

**Table 5:** Logistic regression analysis, shedding light on the determinants of the score of the third part (“attitudes towards recommending anti-HPV vaccination”) for the first and second subsets of items, respectively.

Variable	Odds Ratio	95% CI
**First subset (Hosmer–Lemeshow test, chi-squared = 8.08, *p* = 0.4255)**		
Age	0.99	0.93–1.05
Gender (male)	1.34	0.61–2.93
Clinical ward (pediatrics)	6.40	1.26–32.48
Years of experience (more than 5 years)	1.63	0.37–7.15
Knowledge score	1.13	0.96–1.33
**Second subset (Hosmer–Lemeshow test, chi-squared = 3.53, *p* = 0.8968)**		
Age	0.99	0.94–1.05
Gender (male)	0.51	0.24–1.09
Marital status (married versus not married)	0.60	0.27–1.33
Years of experience (more than 5 years)	0.75	0.17–3.25

## Supplementary Materials

The following is available online at https://www.mdpi.com/2076-393X/7/4/157/s1.
