# Supplementary material for: Knowledge of Human Papillomavirus (HPV), Attitudes and Practices Towards Anti-HPV Vaccination Among Israeli Pediatricians, Gynecologists, and Internal Medicine Doctors: Development and Validation of an Ad Hoc Questionnaire
Source: Vaccines (Basel). 2019 Oct 21;7(4):157. doi: 10.3390/vaccines7040157 (PMC6963669; doi:10.3390/vaccines7040157)
Supplement: Supplementary file 1 [file vaccines-07-00157-s001.pdf]

Knowledge of Human Papillomavirus (HPV), Attitudes and Practices Towards Anti-HPV Vaccination Among Israeli Pediatricians, Gynecologists and Internal Medicine Doctors: Development and Validation of an Ad Hoc Questionnaire

FIRST PART

1. How old are you?
2. Which is your gender? Female / male
3. Which is your religious creed (if any)? Jewish / Christian / Muslim / Druze / other
4. Which is your marital status? Married / not married
5. Which is your profession? Resident / specialist/doctor
6. In which clinical ward do you work? Pediatrics / gynecology / internal medicine

SECOND PART

1. Which is the frequency of gynecological examinations? Once every 6 months / once every 1 year / once every 2 years / check-up only if needed
2. To whom is the Pap test recommended? There are no special recommendations / to every girl aged >18 years / to women aged 25-64 every 3 years / to women aged >45 years every 2 years
3. Which are the preventative strategies for the cervical cancer? Pap test only / HPV vaccine only / Pap test and HPV vaccine / cervical cancer is a disorder that cannot be prevented in any way
4. Which are the Israeli Ministry of Health's guidelines for the anti-HPV vaccination? To offer the anti-HPV vaccine to girls before their first sexual intercourse / to offer the anti-HPV vaccine to girls and boys before their first sexual intercourse / to offer the anti-HPV vaccine only to girls aged 9-12 years / to offer the anti-HPV vaccine to girls and boys aged 9-12 years / to offer the anti-HPV vaccine to the entire population
5. What is the number of existing HPV strains? Only 1 strain / 2 strains / more than 200 strains
6. What is the number of HPV strains related to cervical cancer and other malignancies? Only 1 major strain / 2 major strains / 4 major strains
7. Which are the body regions that can be infected by HPV? Ano-genital / mouth mucosa / skin

8. Against which disease(s) does the anti-HPV vaccination offer protection? Anal cancer / genital warts / HIV / cervical cancer / penile cancer / colorectal cancer / ano-genital cancers and warts
9. Which is the percentage of girls aged 15-18 years being sexually active? 0% / 1-24% / 25-49% / more than 50%
10. Which is the percentage of boys aged 15-18 years being sexually active? 0% / 1-24% / 25-49% / more than 50%

THIRD PART (each item on a likert-scale 1 to 5)

First sub-set

1. I recommend the anti-HPV vaccination to teenage girls.
2. I recommend the anti-HPV vaccination to teenage boys.
3. Anti-HPV vaccination should be routinely administered to females aged 9-12 years.
4. Anti-HPV vaccination should be routinely administered to males aged 9-12 years.
5. In females, the importance of the anti-HPV vaccine is similar to that of other vaccines.
6. In males, the importance of the anti-HPV vaccine is similar to that of other vaccines.
7. The anti-HPV vaccine is optional for females aged 9-12 years.
8. The anti-HPV vaccine is optional for males aged 9-12 years.
9. HPV has severe complications that justifies vaccination.
10. During the anti-HPV vaccination, elements of sexual education should be provided to children.
11. I am not sure the anti-HPV vaccine provides long-term protection.
12. The children get too many vaccinations I do not have time to educate parents about the anti-HPV vaccination.
13. The financial cost of the anti-HPV vaccine prevents me from recommending it.
14. The national guidelines for the anti-HPV vaccination are unclear.
15. The absence of preventive medicine services in some settings prevents me from recommending the anti-HPV vaccine to parents.
16. National guidelines related to the anti-HPV vaccination affect my decision whether to recommend or not the anti-HPV vaccine.
17. I am confident I can address parents' questions and concerns about the anti-HPV vaccine when vaccinating their female children.
18. I am confident I can address parents' questions and concerns about the anti-HPV vaccine when vaccinating their male children.

19. I am confident that I can deal with the parental concern that, following the anti-HPV vaccination, their female child's sexual activity will increase.
20. I am confident that I can deal with the parental concern that, following the anti-HPV vaccination, their male child's sexual activity will increase.
21. I am confident that I can deal with parental concerns about the side effects of the anti-HPV vaccine that may occur in their female children.
22. I am confident that I can deal with parental concerns about the side effects of the anti-HPV vaccine that may occur in their male children.
23. I have enough time to identify and address the reasons for parental refusal of the anti-HPV vaccine for their female children.
24. I have enough time to identify and address the reasons for parental refusal of the anti-HPV vaccine for their male children.
25. I think I have a great influence on the parents' decision whether to vaccinate or not their female children against HPV.
26. I think I have a great influence on the parents' decision whether to vaccinate or not their male children against HPV.
27. Usually I can convince parents who hesitate to vaccinate their female children.
28. Usually I can convince parents who hesitate to vaccinate their male children.
29. When parents have concern about the anti-HPV vaccine and refuse to vaccinate their female child, I think I am not able to change their minds.
30. When parents have concern about the anti-HPV vaccine and refuse to vaccinate their male child, I think I am not able to change their minds.

Second-subset

31. I talk frequently about sexually transmitted diseases with female adolescents when they visit my clinic.
32. I talk frequently about sexually transmitted diseases with male adolescents when they visit my clinic.
33. I explain to adolescents that the anti-HPV vaccine is safe.
34. I provide adolescents with printed material and detailed information about the anti-HPV vaccine.
35. In case of concerns, I always ask which are the major sources of doubts.
36. In case of concerns, I tell parents that, as parent, I have vaccinated my child/children against HPV.
37. In case of concerns or doubts, I push parents to vaccinate their children against HPV.
38. In case of concerns or doubts, I generally suggest postponing the anti-HPV vaccination.

39. I provide parents with printed material specifically dealing with their concerns.
40. I provide parents with information about the anti-HPV vaccination.
41. Before the vaccination, I provide parents with written information adapted to their socio-cultural background.
42. I administer questionnaires to parents to identify their concerns.
43. I provide parents with friendly explanations using interactive aids (such as a computer or a smart phone app).
